# Lung cancer in older patients with granulomatosis with polyangiitis: a report of three cases

**DOI:** 10.1186/s12890-024-03024-7

**Published:** 2024-04-22

**Authors:** Malgorzata Potentas-Policewicz, Malgorzata Szolkowska, Katarzyna Blasinska, Dariusz Gawryluk, Malgorzata Sobiecka, Justyna Fijolek

**Affiliations:** 1 Geriatrics Department, Dr Anna Gostynska Wolski Hospital, Warsaw, Poland; 2 Department of Pathology, National Tuberculosis and Lung Diseases Research Institute, Warsaw, Poland; 3 Department of Radiology, National Tuberculosis and Lung Diseases Research Institute, Warsaw, Poland; 4 The Third Department of Pneumonology and Oncology, National Tuberculosis and Lung Diseases Research Institute, Warsaw, Poland; 5 The First Department of Pneumonology, National Tuberculosis and Lung Diseases Research Institute, Warsaw, Poland

**Keywords:** Carcinoma, Case report, Granulomatosis with polyangiitis, Lung cancer, Older patients

## Abstract

**Background:**

Granulomatosis with polyangiitis (GPA) is characterized by necrotizing granulomatous inflammation with necrotizing vasculitis predominantly affecting small to medium vessels. The survival rates have drastically improved; however, GPA can be lethal, with older patients having a worse prognosis and higher mortality than younger patients. Moreover, the incidence of various cancers has been reported to increase in patients with GPA. We aimed to discuss possible associations between GPA and lung cancer and emphasize the associated diagnostic challenges.

**Case presentation:**

We encountered three older patients with chronic GPA who developed lung cancer during long-term follow-up. Two of the patients had a smoking history, with one having silicosis and the other having chronic obstructive pulmonary disease. Furthermore, all of them had radiation exposure from repeated radiography/computed tomography. All the patients had confirmed GPA, and vasculitis relapse was first suspected when new lung lesions were noted during follow-up. However, they had no new clinical symptoms, and serum ANCA titer increased only in one patient. All the patients received standard immunosuppressive treatment but eventually died.

**Conclusions:**

Lung cancer is uncommon in patients with GPA; however, the similarity between the imaging findings of lung cancer and GPA may pose a diagnostic challenge. Clinicians should be particularly vigilant when treating older patients with an increased risk of cancer, as they are often asymptomatic or have poorly apparent clinical features.

## Background

Granulomatosis with polyangiitis (GPA) is characterized by necrotizing granulomatous inflammation with necrotizing vasculitis predominantly affecting small to medium vessels. This condition belongs to the antineutrophil cytoplasmic antibody (ANCA)-associated vasculitis (AAV) family, because circulating ANCAs are detected in most patients [1]. Despite advances in treatment, GPA can be lethal, with older patients having a worse prognosis and higher mortality than younger patients [2]. Patients with AAV have a high incidence of cancer, specifically non-melanoma skin cancer, leukemia, and bladder cancer, which may be associated with cyclophosphamide (CYC) use [3]. Lung cancer is uncommon in patients with GPA; however, the similarity between the imaging findings of lung cancer and GPA may pose a diagnostic challenge and delay diagnosis. Diagnosis may be particularly difficult in patients with confirmed chronic GPA, in whom lung cancer may mimic a relapse of vasculitis. We present three rare cases of lung cancer in older patients with chronic GPA, and discuss the potential association between these two conditions and the associated diagnostic challenges.

## Case presentation

**Fig. 1:**
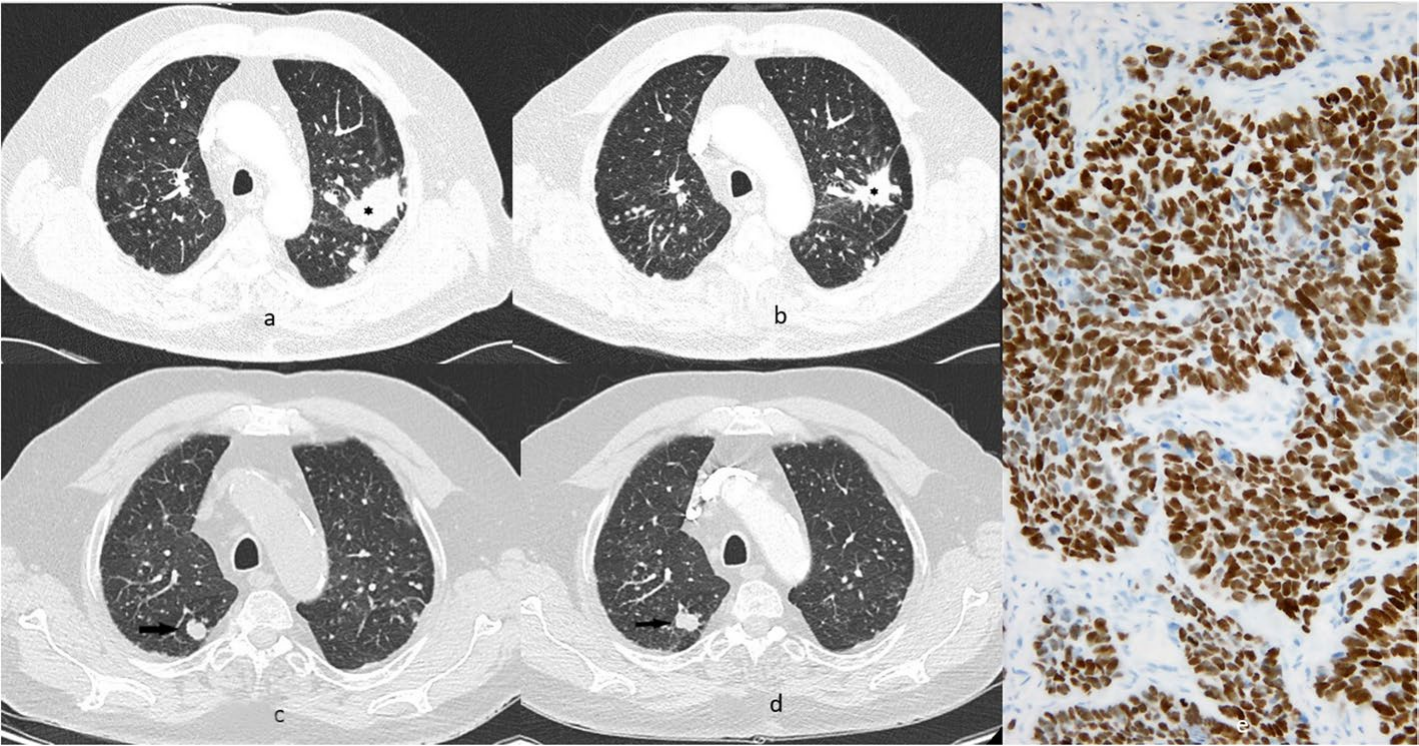
Patient 1. Chest CT, lung window, axial plane; histological examination. **a** Baseline study. Nodules in the left lung, the larger lesion located in segment 1 + 2 of the upper lobe (black asterisk). The subsequent examination after one year (**b**) shows partial regression of lesions. Another chest CT during the follow -up (**c**) revealed a new nodule in the right upper lobe which enlarged at 3-month observation (black arrow) (**d**). **e** Microscopic image of poorly-differentiated squamous cell carcinoma in resection specimen (A—hematoxylin and eosin stain, magnification × 200; B—cytokeratin AE1AE3 immunohistochemical reaction, magnification × 200)

Patient 1 was diagnosed with GPA in February 2012 at 69 years of age. He was an ex-smoker, had a history of silica exposure, and had undergone basal cell carcinoma surgery several years previously. GPA was diagnosed based on clinical symptoms and ANCA-positive status. The patient presented with peripheral polyneuropathy, skin lesions, and hematuria. Chest computed tomography (CT) revealed lung nodules without cavitation (Fig. 1a). Standard immunosuppressive treatment was administered; however, due to incomplete regression of lung lesions after 6 months, the patient underwent a surgical lung biopsy. Histopathological examination revealed silicosis and confirmed diagnosis of GPA. The treatment was completed in December 2013 (Fig. 1b) (total CYC dose: 31.5 g); however, in January 2014, the patient was readmitted because of a GPA flare-up. At that time, he experienced weakness, dyspnea, and hemoptysis. Chest CT revealed progression of lung nodules, and bronchofiberoscopy revealed multiple bronchial ulcerations. Urinalysis revealed a recurrence of microscopic hematuria, and the ANCA titer was increased. Histological examination of bronchial specimens confirmed the diagnosis of GPA. Rituximab was administered, resulting in clinical improvement and ANCA negativity. The next vasculitis flare-up occurred in April 2015. Rituximab was restarted, and oral methotrexate was administered as maintenance therapy. The patient was followed up every 6 months, and in August 2017 (when the patient was aged 74 years), CT revealed a new nodule in the right upper lobe (Fig. 1c). A second GPA relapse was suspected; however, ANCAs were not detected, and the patient was asymptomatic. The next CT, performed 3 months later, revealed that the nodule had grown (Fig. 1d). Bronchofiberoscopy results (cultures and cytology examination) were negative, and the patient underwent thoracotomy. Due to significant impairment of lung function, the wedge resection of the nodule was performed. Histological examination revealed squamous cell carcinoma (stage T2aN0R0L0V0) (Fig. 1e). The patient received radiotherapy (6000 cGy/t). Despite this, he died 14 months later because of cancer progression.

**Fig. 2:**
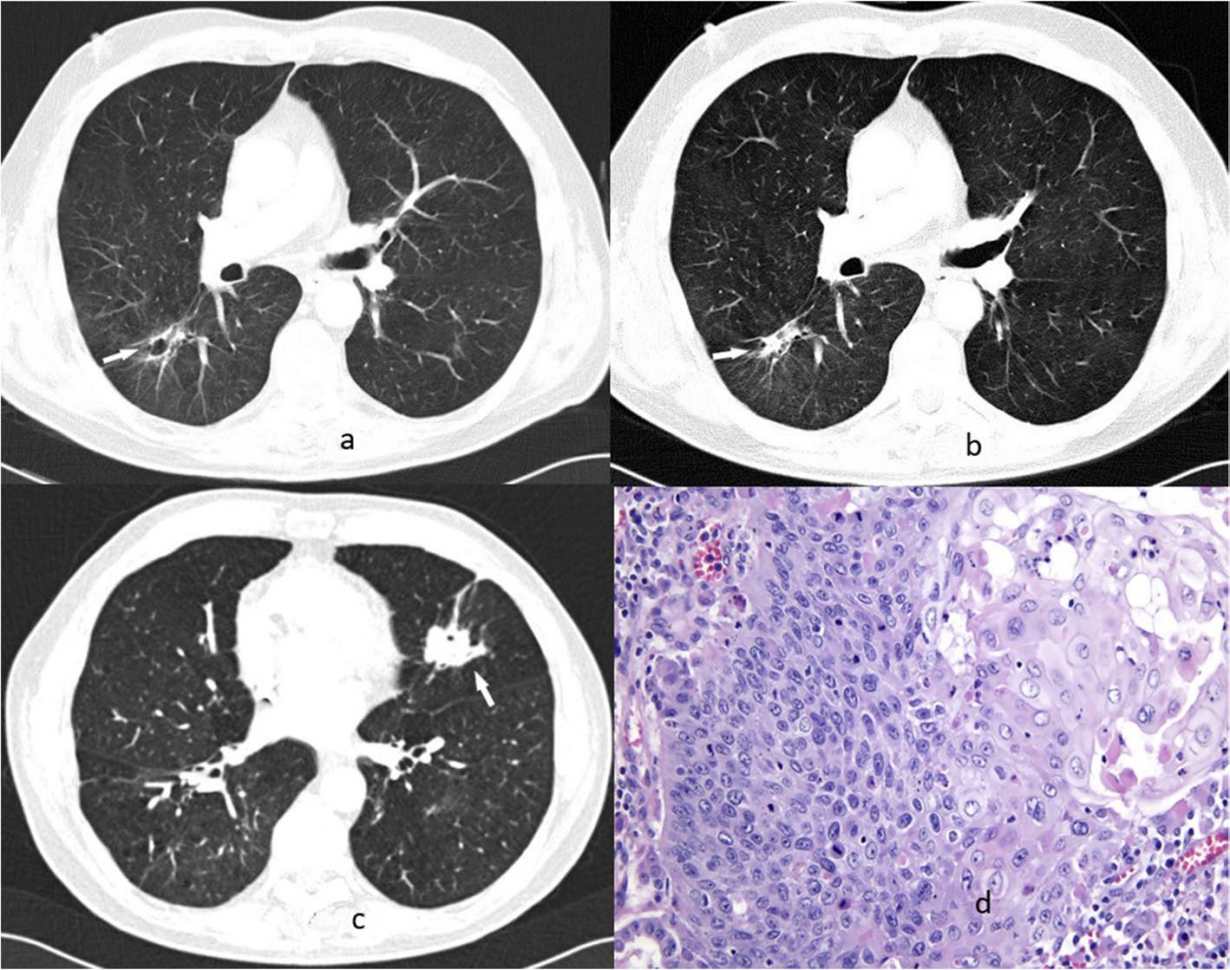
Patient 2. Chest CT, lung window, axial plane; histological examination. **a** Baseline study showed the cavitated lesion in segment 6 of the right lower lobe (white arrow) which decreased in the next examination (**b**). A follow-up examination 5 years later showed a new lesion with cavitation in the left upper lobe (white arrow) (**c**). **d** Microscopic image of keratinizing squamous cell carcinoma. Resection specimen (hematoxylin and eosin stain, magnification × 200)

Patient 2 was diagnosed with GPA in March 2006 at 61 years of age. He had a smoking history, and had been diagnosed with chronic obstructive pulmonary disease (COPD) 5 years previously. GPA was diagnosed based on clinical symptoms and ANCA-positive status, and confirmed via nasal mucosal biopsy. The patient presented with sino-nasal symptoms and kidney involvement. Chest CT revealed multiple lung nodules, some of which exhibited cavitation (Fig. 2a). The patient received standard immunosuppressive therapy from March 2006 to May 2007 and showed improvement (Fig. 2b); however, in November 2008, he was readmitted because of a GPA flare-up, presenting with diffuse alveolar hemorrhage. Standard immunosuppressive treatment (total CYC dose: 54 g) was restarted and continued until September 2010, resulting in symptom resolution, improvement in lung lesions, and ANCA titer reduction. In May 2015 (when the patient was aged 70 years), CT performed during a follow-up visit revealed a single new tumor with cavitation in the left lung (Fig. 2c). The patient had no symptoms, and laboratory test results showed a significant increase in ANCA titer. This pattern of findings was different from that of GPA, and histological examination of the material obtained via bronchofiberoscopy revealed squamous cell carcinoma (stage pT2aN0M0R0L0V0). The patient underwent a left upper lobectomy (Fig. 2d shows the microscopic image of resected specimen). However, he died of cancer progression 4 years later.

**Fig. 3:**
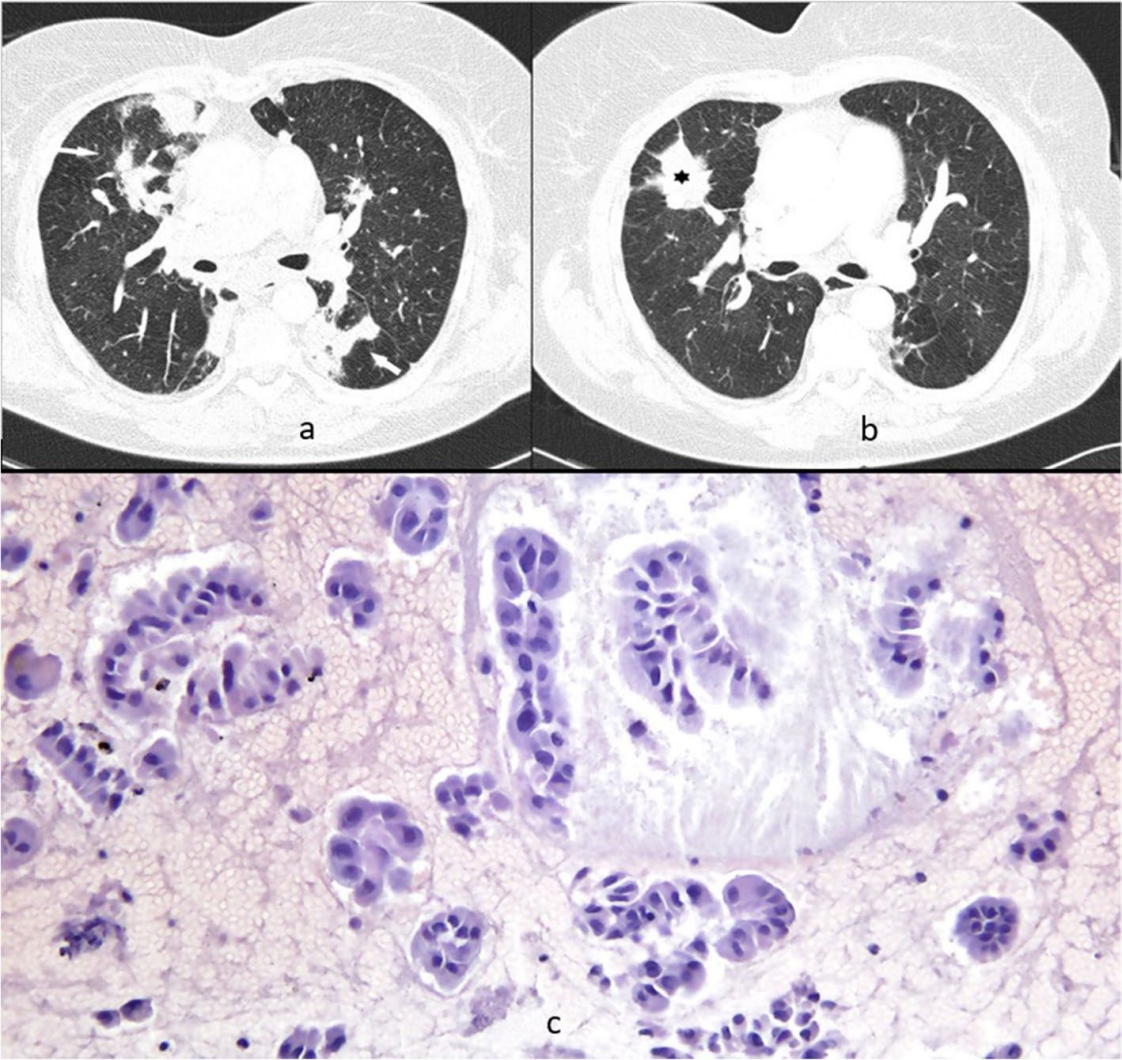
Patient 3. Chest CT, lung window, axial plane; histological examination. **a** Baseline CT revealed multiple nodules in both lungs (white arrows). **b** A follow-up CT scan after 7 years showed new solid lesion in the right lung (black asterisk). **c** Microscopic image of adenocarcinoma in cell block (hematoxylin and eosin stain, magnification × 200)

Patient 3 was diagnosed with GPA in February 2005 at 71 years of age. She had never smoked, but had diabetes, hypertension, and bronchial asthma. The disease was limited to the lungs (chest CT revealed multiple lung nodules) (Fig. 3a) and was confirmed based on histological examination findings and the patient’s ANCA-positive status. The patient was treated with standard immunosuppressive drugs (total CYC dose: 58.5 g) until March 2007, and showed clinical and radiological improvements. In February 2014 (when the patient was aged 80 years), a chest CT revealed a single new tumor in the right lung (Fig. 3b). Relapse of GPA was considered; however, ANCA test results were negative, and the patient had no new symptoms. Biopsy revealed lung adenocarcinoma (clinical stage T2aN0M0) (Fig. 3c). Due to the clinical burden and lung function limitations (persistent airway obstruction and desaturation in a 6-min walk test), the patient was ineligible for surgical treatment. She received palliative care and died 1 year later.

## Discussion and conclusions

This report presents three older patients with GPA who developed lung cancer during long-term follow-up. These cases highlighted several important issues: 1) patients with GPA should undergo long-term monitoring, even if they are in remission; 2) histological examination should be performed in patients with GPA who develop new lung lesions, especially in the absence of other organ involvement and signs of vasculitis; and 3) in patients with GPA, particularly older patients, oncological vigilance should be maintained. Moreover, these cases prompted us to consider the possible association between GPA and lung cancer, and to emphasize the associated diagnostic challenges.

Although many studies reported an increased incidence of cancers in AAV compared to the general population [3–7], data on lung cancer in patients with GPA are limited. Chemouny et al. [8] described five patients with AAV who had lung cancer developing concurrently or within 2 years. Most of these patients (4/5) were elderly and ANCA positive and all of them had renal involvement. Three patients were diagnosed with squamous cell carcinoma, and two with adenocarcinoma. Masiak et al. [9] reported the case of a 63-year-old man who developed small-cell lung cancer 12 years after GPA diagnosis. The patient had chronic GPA and during the course of the disease, relapses were observed with progression and cavitation of the infiltrates in the lungs. The patient was hospitalized because of headaches, general weakness, decrease in exercise capacity, and a tear of the eye, which combined with the presence of the new infiltrate in the lung, was suggestive of the next relapse of vasculitis, but finally small-cell lung cancer was diagnosed. In turn,Toriyama et al. [10] presented a case of lung cancer that developed during long-term CYC treatment for GPA, while Andino et al. [11] described a 50-year-old male with chronic renal failure secondary to GPA, who 23 years after the diagnosis of vasculitis (and 12 years after the completion of treatment) developed multifocal lung adenocarcinoma with pleural effusion. Herein, we described three patients with GPA who developed lung cancer 5–9 years after being diagnosed with vasculitis. All three patients were aged above 60 years when GPA was diagnosed, and all lung cancers were detected when GPA was in clinical remission. Increases in lung cancer incidence in patients with GPA have been reported [6–8]; however, only a single Korean study demonstrated a significant increase in the adjusted hazard ratio (HR) of lung cancer (HR = 1.92, 95% confidence interval, 1.20–3.07) [12]. In contrast, a retrospective analysis of the French Vasculitis Study registry found that lung cancer was the most frequent cause of death due to malignancy in patients with systemic necrotizing vasculitides [13]. Therefore, patients with GPA must be monitored over the long term, even during remission, as our cases strongly highlight.

The classical presentation of GPA includes upper respiratory tract, lung, and kidney symptoms [14]. Lung findings are non-specific; however, the most common CT features include nodules and masses, often with cavitation [15]. These features require differentiation, mainly from those of infections and primary and secondary lung malignancies. Diagnosis is particularly challenging when lesions are limited to the lungs [16]; moreover, because both GPA and lung cancer have similar clinical features, symptom masking may occur [9]. Finally, lung cancer may coexist with GPA [17]; this should be considered when diagnosing patients with AAV and pulmonary lesions. Our patients had confirmed GPA, and a relapse of vasculitis was first suspected when new lung lesions were noted during follow-up. However, they had no new clinical symptoms, and serum ANCA titer increased only in patient 2. Additionally, patient 1. developed numerous nodular lesions owing to silicosis, which further complicated clinical interpretation of that patient’s CT scans. Owing to diagnostic uncertainty, all our patients underwent a biopsy, which allowed us to establish the correct diagnosis. Our cases strongly highlight that long-term remission of vasculitis does not eliminate the need for vigilance when assessing new lung lesions in patients with GPA. Such patients should be further investigated and not automatically diagnosed with vasculitis relapse. Biopsy and histological examinations should be considered, as they are essential for proper differential diagnosis.

The association between GPA and lung cancer has yet to be fully established. Systemic autoimmune rheumatic diseases are associated with an increased risk of cancer, with the overall cancer risk for most patients being the highest in the first year of follow-up and decreasing thereafter [18]. In contrast, in our patients, lung cancer developed late in the observation period (5, 9, and 9 years after vasculitis diagnosis respectively). Several mechanisms may contribute to increased cancer risk in patients with autoimmune rheumatic diseases, including chronic inflammation and damage from the disease, cytotoxic therapies, and inability to clear oncogenic infections [19]. To the best of our knowledge, detailed studies on lung cancer formation in patients with vasculitis have not been conducted. As lung parenchymal inflammation is a symptom of GPA, the possible contribution of chronic inflammation to cancer development cannot be ruled out. The impact of CYC has also been considered. In fact, an increased incidence of non-melanoma skin cancers, bladder cancer and myeloid leukemia was demonstrated among patients exposed to cumulative CYC doses > 36 g [7]; however, lung injury after CYC treatment is rare (< 1%) and mainly manifests as early-onset pneumonitis or late-onset lung fibrosis [20]. In the study analysing solid malignancies among patients with Wegener’s granulomatosis treated with etanercept (WGET), patients who developed solid malignancies were more likely to be enrolled into the trial with a disease relapse (85% versus 54%, p = 0.04), and had longer disease duration (6.4 ± 3.9 years versus 3.3 ± 4.8 years, p = 0.001), while there were no differences in the assigned treatment, age, gender, or extent of disease [21]. On the other hand, the report of case of GPA that lung cancer has developed during the long-term treatment with CYC also exists. In that patient, cumulative dose of CYC was about 200 g and the duration was 12 years, which could be the risk of carcinogenesis [10]. Notably, rituximab treatment is not associated with an increased malignancy risk compared with the general population and could therefore be a safe alternative to CYC in the treatment of AAV [22]. Other factors that may be considered include environmental factors such as cigarette smoking, radiation, and occupational lung carcinogens, with the common risk factors for both diseases can be exposure to silica and infectious agents [8, 23]. There is a hypothesis that there is a common pathway between AAV and malignancy, namely, that patients with vasculitis have an intrinsically higher risk of developing malignancies based on a state of acquired immunological dysfunction underlying both diseases [12, 24]. The observed ANCA titer variations related to lung cancer evolution may be also indicative of a link between both diseases, decreasing or becoming undetectable after cancer treatment and increasing with cancer relapse [8]. Of the three our patients, two had a smoking history, with one of them having silicosis and the other having COPD. Furthermore, all patients were exposed to radiation because they repeatedly underwent radiography/CT. However, only in one patient (patient 2) the ANCA titer was increased at the time of lung cancer diagnosis, while in other ANCA was not detected. Another factor is older age of the patients. Recent data have shown that the incidence of cancer strongly increases after the age of 50 years; this has been thought to result from reduced immune system function, leading to inefficient purging of dysfunctional cells and an increase in the number of altered cells (including pre-neoplastic cells) [25]. According to data from NCI’s Surveillance, Epidemiology, and End Results Program, the median age of cancer diagnosis is 66 years, with the median age for lung cancer being 71 years [26]. Interestingly, our preliminary analysis of the data of 300 patients with GPA observed since 1978 (data not published) revealed that none of the younger patients were diagnosed with lung cancer, whereas among the older patients (accounting for approximately 15% of all the groups), three developed lung cancer; the cases of these three patients have been reported herein, with the patients being 74, 70, and 80 years old at the time of cancer diagnosis. In conclusion, older age is an important risk factor for lung cancer, and oncological vigilance should be strongly maintained in patients with GPA, especially older patients, who develop new lung symptoms.

In summary, we present three cases of older patients with GPA who developed lung cancer during long-term follow-up. These cases demonstrate that patients with GPA should be monitored even when vasculitis is in remission. This strategy enables early detection and appropriate treatment of cancer. Additionally, we highlight the diagnostic challenges in patients with GPA who develop new lung lesions, emphasizing that although relapse is considered first, clinicians must suspect other potential causes, such as malignancy. In such cases, biopsy and histological examination are pivotal for appropriate differential diagnosis. Finally, clinicians should be particularly vigilant when managing older patients with GPA, who face a higher risk of cancer than younger patients. Our cases raise the question of a potential link between vasculitis and lung cancer and highlight the clinical conditions recurrently observed together; however, they do not allow conclusions regarding the significance of their association or pathophysiological link, and further studies on a larger number of patients are needed. Nonetheless, physicians should be aware of this potential association and consider it when treating patients with AAV who have new pulmonary lesions.

## Data Availability

The authors confirm that the data supporting the findings of this study are available within the article.

## Abbreviations

AAV: Antineutrophil cytoplasmic antibody-associated vasculitis
ANCA: Antineutrophil cytoplasmic antibody
COPD: Chronic obstructive pulmonary disease
CT: Computed tomography
CYC: Cyclophosphamide
GPA: Granulomatosis with polyangiitis
HR: Hazard ratio
